# Epigenetic Changes Modulate Schistosome Egg Formation and Are a Novel Target for Reducing Transmission of Schistosomiasis

**DOI:** 10.1371/journal.ppat.1004116

**Published:** 2014-05-08

**Authors:** Vitor Coutinho Carneiro, Isabel Caetano de Abreu da Silva, Eduardo José Lopes Torres, Stephany Caby, Julien Lancelot, Mathieu Vanderstraete, Silviya D. Furdas, Manfred Jung, Raymond J. Pierce, Marcelo Rosado Fantappié

**Affiliations:** 1 Instituto de Bioquímica Médica, Programa de Biologia Molecular e Biotecnologia, Universidade Federal do Rio de Janeiro, Rio de Janeiro, Brasil; 2 Universidade do Estado do Rio de Janeiro, Faculdade de Ciências Médicas, Rio de Janeiro, Brasil; 3 CIIL, INSERM U1019 – CNRS UMR 8204, Université Lille Nord de France, Institut Pasteur de Lille, Lille, France; 4 Institute of Pharmaceutical Sciences, Albert-Ludwigs-University, Freiburg, Germany; George Washington University School of Medicine and Health Sciences, United States of America

## Abstract

Treatment and control of schistosomiasis relies on the only available drug, praziquantel, and the search for alternative chemotherapeutic agents is therefore urgent. Egg production is required for the transmission and immunopathology of schistosomiasis and females of *S. mansoni* lay 300 eggs daily. A large fraction of the total mRNA in the mature female worm encodes one eggshell protein, Smp14. We report that the nuclear receptors SmRXR1 and SmNR1 regulate *Smp14* transcription through the recruitment of two histone acetyltransferases (HATs), SmGCN5 and SmCBP1. The treatment of HEK293 cells with histone deacetylase (HDAC) inhibitors (NaB or TSA) produced an 8-fold activation of the SmRXR1/SmNR1-mediated *Smp14* promoter activity. Incubation with synthetic HAT inhibitors, including PU139, significantly impaired the *Smp14* promoter activity in these cells. Worm pairs cultivated in the presence of PU139 exhibited limited expression of Smp14 mRNA and protein. ChIP analysis demonstrated chromatin condensation at the *Smp14* promoter site in worms treated with PU139. ChIP also revealed the presence of H3K27me3 and the absence of RNA Pol II at the *Smp14* promoter region in the PU139-treated worms. Most significantly, the PU139-mediated inhibition of *Smp14* expression resulted in a significant number of abnormal eggs as well as defective eggs within the ootype. In addition, scanning electron microscopy revealed structural defects and unformed eggshells, and vitelline cell leakage was apparent. The dsRNAi-targeting of SmGCN5 or SmCBP1 significantly decreased *Smp14* transcription and protein synthesis, which compromised the reproductive system of mature female worms, egg-laying and egg morphology. Our data strongly suggest that the inhibition of *Smp14* expression targeting SmGCN5 and/or SmCBP1 represents a novel and effective strategy to control *S. mansoni* egg development.

## Author Summary

*Schistosoma mansoni* is a parasitic worm that causes schistosomiasis, a debilitating disease in Africa and South America. Female worms mated with males produce hundreds of eggs that can reach the environment to propagate the biological cycle, or become trapped in host tissues, triggering inflammation and pathology. Because eggshell formation is a key step in egg development and viability, we have studied the molecular mechanisms of *S. mansoni* eggshell development, focusing on a major eggshell gene, *Smp14*. Using a variety of technical and biological approaches, we obtained strong evidence that eggshell formation depends on nuclear receptors and coactivators with chromatin modifying activities, mainly histone acetylation. Inhibition or partial deletion of *S. mansoni* histone acetyltransferases impaired the expression of Smp14, culminating in a severe negative effect on eggshell formation. Our findings will contribute not only to a better understanding of sex and tissue-specific gene regulation in *S. mansoni* but also provide an alternative strategy for interfering with the egg production, which might be targeted in novel therapeutics directed against this parasite.

## Introduction

Schistosomes are large metazoan pathogens that parasitize over 200 million people worldwide, resulting in up to 300,000 deaths per year [1]. Praziquantel is the only drug available, and despite its efficacy, it does not prevent re-infection, it is not effective against juvenile schistosomes, its mechanism of action has not been elucidated, and most importantly, resistance to this drug is already a concern [2]. The search for new drug targets is critical for developing novel strategies to combat this major pathogen. Sexually mature adult female *S. mansoni* lay a large number of eggs daily, which are responsible for both the transmission and pathogenesis of schistosomiasis [3]; therefore, understanding the mechanisms involved in schistosome reproductive biology is of particular interest. Because eggshell formation is a key step for determining the quality and quantity of eggs laid [4], we focused our studies on the molecular mechanisms of *S. mansoni* eggshell development. The major *S. mansoni* eggshell protein is Smp14 [5]–[7]. The *Smp*14 gene is exclusively expressed in mature vitelline cells in the vitelline duct and within the egg, enclosed by the eggshell, inside the ootype [4]. *Smp14* is the most abundant mRNA transcript in sexually mature females and accounts for 10% of the mRNA of the entire organism [8]. Our previous research suggested that *Smp14* transcription is regulated by the nuclear receptor heterodimer SmRXR1/SmNR1, which recruits coactivators with histone modifying (acetylation and methylation) activities [9]–[14]. We previously demonstrated the *in vitro* assembly of the SmRXR1/SmNR1 heterodimer and the two major histone acetyltransferases (HATs) in *S. mansoni*, SmGCN5 and SmCBP1, on a DNA response element within the *Smp14* promoter region [14]. In addition, the physical interactions between SmRXR1, SmNR1, SmGCN5 and SmCBP1 have been mapped [13]. Importantly, we have demonstrated that SmGCN5 is localized on the transcriptionally active chromatin within the mature vitelline cells of female parasites [12]. In this study, we extended our original findings by characterizing, *in vivo*, the role of SmGCN5 and SmCBP1 in the transcriptional regulation of *Smp14*. We demonstrated that histone acetylation and chromatin remodeling by both schistosome HATs are essential for the activation of *Smp14*. More significantly, the inhibition of the histone acetyltransferase activity of SmCBP1 or SmGCN5 by a HAT inhibitor or RNA interference decreased *Smp14* transcription and was associated with a severe negative effect on eggshell formation. Taken together, our data strongly suggest that histone acetyltransferase activity can represent a possible target for limiting parasite transmission, i.e., by decreasing the number of viable eggs deposited in the environment and reducing the production of the soluble egg and eggshell antigens involved in the immunopathogenesis of schistosomiasis.

## Results

### *Smp14* transcriptional activation is dependent on histone acetylation

**Figure 1:**
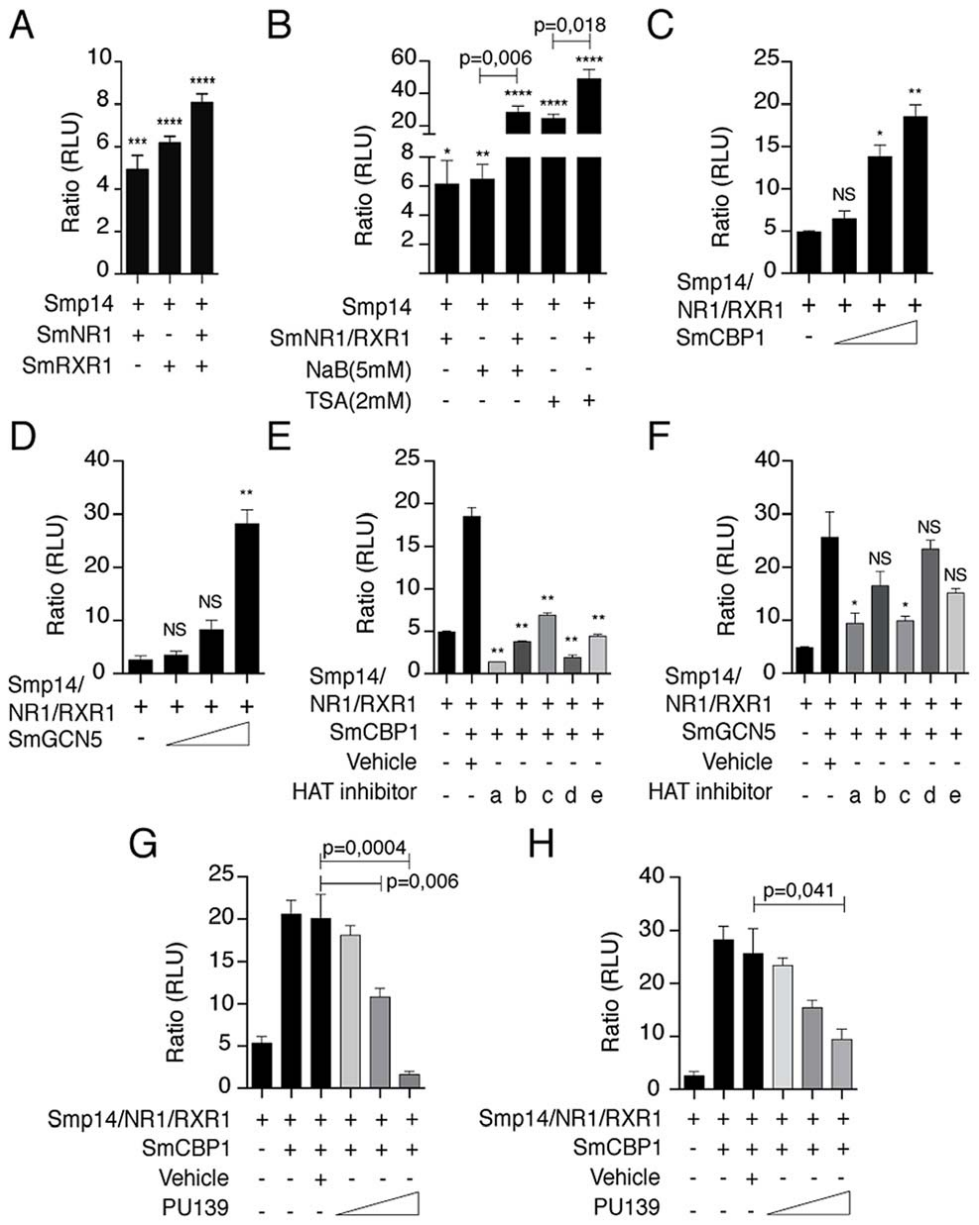
*Smp14* promoter-dependent activation by the SmRXR1/SmNR1 heterodimer and the HATs SmGCN5 and SmCBP1. The luciferase gene reporter assays were performed in HEK293 cells. The *Smp14* promoter-target sequence was cloned upstream of the luciferase reporter gene in the vector pGL4.23 (*Smp14*/3X-pGL4.23). This vector was co-transfected with the SmRXR1/SmNR1 heterodimer and/or co-transfected with SmCBP1 or SmGCN5 (A–H). The HDAC inhibitors NaB (5 mM) and TSA (2 µM) were tested (B). The dose-dependent transcriptional activation of the *Smp14* promoter by SmCBP1 or SmGCN5 is shown (C and D). Small synthetic HAT inhibitors (2 µM) were tested in the transfected cells (E and F): PU139 (a), PU141 (b), SF7 (c), SF18 (d) and SF19 (e). Dose-dependent inhibition (0.5 nM, 1 µM and 2 µM) of SmCBP1 and SmGCN5 by the most potent inhibitor, PU139 (G and H). Results were plotted in relation to the firefly luciferase activity obtained from cells transfected with the *Smp14*/3X-pGL4.23 vector alone. Graphs are pooled from three independent experiments. Student's t-test was applied, with *p<0.05, **p<0,01, ***p<0.001 and ***p<0.001. Western blot data are representative of three independent experiments.

First, we investigated whether the SmRXR1/SmNR1 heterodimer activated the transcription of a reporter gene under the control of the *Smp14* promoter. Because schistosome cell lines are not available, we used HEK293 cells as a surrogate system to investigate *Smp14* transcriptional regulation by schistosome nuclear receptors. We observed a SmRXR1/SmNR1-dependent transcriptional activation of the *Smp14* promoter (Fig. 1A). Because transcriptional activation is generally correlated with histone acetylation, we used classical histone deacetylase (HDAC) inhibitors (NaB or TSA) to demonstrate that histone acetylation indeed plays a key role in the *Smp14* transcriptional activation by SmRXR1/SmNR1 (Fig. 1B). To further define the role of histone acetylation in the transcriptional activation of the *Smp14* promoter by the nuclear receptor-coactivator complex, expression vectors for the *S. mansoni* HATs SmGCN5 and SmCBP1 were co-transfected into HEK293. As shown in Fig. 1C and D, SmGCN5 and SmCBP1 significantly enhanced the transcriptional activation of the *Smp14* promoter in a dose-dependent manner. To determine, *in vivo*, the assembly of this transcriptional complex on the *Smp14* promoter, we performed ChIP assays with the chromatin extracted from adult worm pairs and antibodies directed against SmRXR1, SmNR1, SmGCN5, SmCBP1 or acetylated H3 (a marker of open chromatin). As seen in Fig. S1, the *Smp14* promoter was immunoprecipitated along with the nuclear receptors and HAT coactivators. These data strongly suggest that *in vivo*, SmGCN5 and SmCBP1 are recruited to the *Smp14* promoter through the stable interaction with SmRXR1/SmNR1, and acetylate histone H3.

HAT inhibition by small synthetic pyridoisothiazolones has been previously investigated [15]. In this study, these potential HAT inhibitors were evaluated for the inhibition of the human HAT subtypes GCN5, p300 and CBP *in vitro* using an immobilized histone peptide substrate and a heterogeneous assay format. The quantification of acetylation was measured using anti-acetyl lysine antibodies and a europium-labeled secondary antibody. Time-resolved fluorescence was used to determine the europium concentration, which is an indication of the degree of histone acetylation. Whereas the N-aryl derivatives PU139 and SF7 acted as broad-spectrum HAT inhibitors, the N-benzyl congener PU141 was selective for the closely related HAT subtypes CBP and p300 (Table S1). SF18 and SF19 demonstrated moderate selectivity for PCAF (Table S1). Because the targets of these inhibitors are the HAT catalytic domains and these enzyme domains are largely conserved across species, we performed additional receptor-reporter assays to assess the effects of five potential synthetic HAT inhibitors on the SmRXR1/SmNR1-, SmGCN5- or SmCBP1-mediated transcriptional activity of the *Smp14* promoter. All five compounds significantly inhibited the activity of SmCBP1; compound “a” (PU139) demonstrated the greatest inhibitory activity (Fig. 1E). When the SmGCN5-mediated transcriptional activity was investigated, only compounds “a” (PU139) and “c” (SF7) were effective, and the PAN-HAT inhibitor PU139 demonstrated the most effective inhibition of HAT (Fig. 1F). A clear dose-dependent inhibition of the transcriptional activity of the *Smp14* promoter by PU139 was observed in HEK293 cells when SmCBP1 and SmGCN5 were co-transfected (Fig. 1G and H).

### HAT inhibition halts *Smp14* transcription and translation in *S. mansoni*

**Figure 2:**
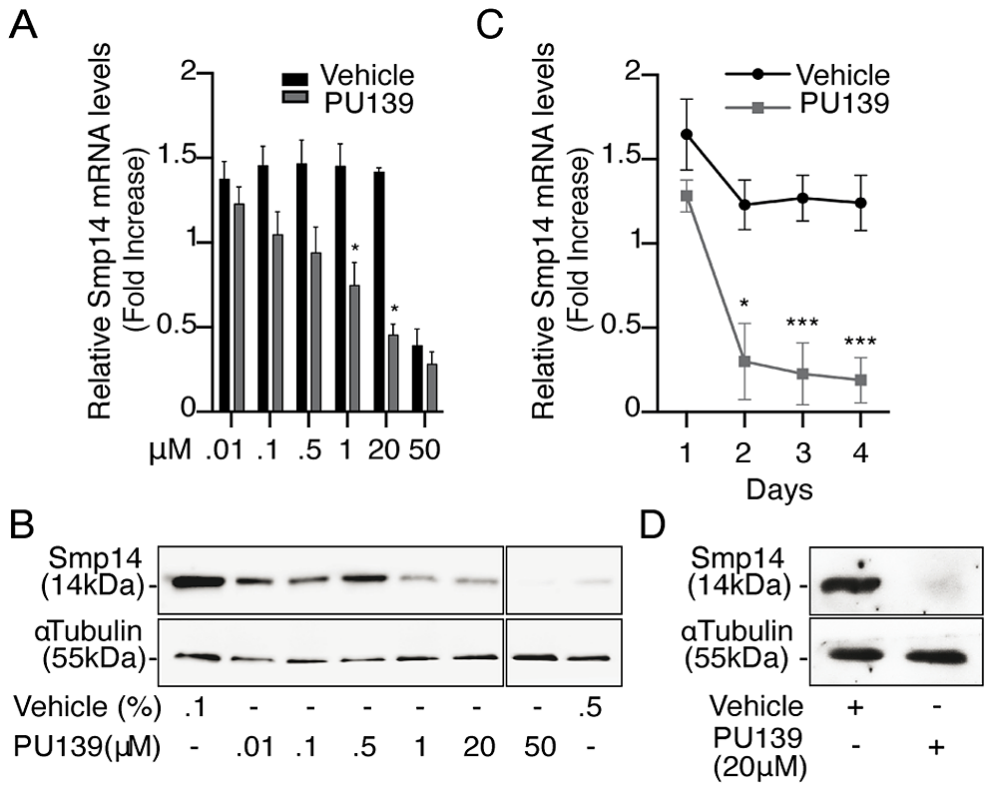
HAT inhibition impairs *Smp14* transcription and translation in *S. mansoni* adult worms. Ten adult worm pairs were cultivated with increasing concentrations of the HAT inhibitor PU139 or DMSO as vehicle. PU139 concentrations between 0.01 and 20 µM were solubilized in 0.1% DMSO, and 50 µM PU139 was only soluble in 0.5% DMSO. (A) qRT-PCR analysis of Smp14 mRNA levels after a two-day incubation time. (B) Western blot analysis of total protein extract from a two-day worm pair culture using polyclonal antibodies against Smp14 (upper panels) and monoclonal antibodies against α-tubulin (lower panels) as loading control. (C) qRT-PCR of *Smp14* after a longer period (four days) of PU139 (20 µM) treatment. (D) Smp14 protein levels at the fourth day of treatment with 20 µM PU139. Graphs are pooled from three independent experiments. Student's t-test was applied, with *p<0.05 and ***p<0.001. Western blot data are representative of three independent experiments.

To confirm that histone acetylation is a prerequisite for *Smp14* transcriptional activation in *S. mansoni*, adult worm pairs were cultivated for two days in the presence of increasing concentrations of PU139 (Fig. 2A). At concentrations that ranged from 1 to 20 µM, PU139 significantly blocked both the transcription (Fig. 2A) and translation (Fig. 2B) of *Smp14*. Furthermore, when the worms were treated for a longer period (3–4 days), the mRNA levels were decreased by the same amount as observed following the two-day incubation (Fig. 2C). Incubation with PU139 for a longer period practically abolished the Smp14 protein levels in the worms (Fig. 2D; compare the two-day incubation period (panel B) with the four-day incubation period (panel D). Although the 20 µM (0.1% DMSO) PU139 treatment was not overtly toxic to the worms (they remained paired and exhibited normal motility, data not shown), increasing the PU139 exposure concentration to 50 µM (0.5% DMSO) was harmful to the parasites (indicated by the separation of the male and female worms, data not shown), and the expected reduction in Smp14 expression was observed [16] (Fig. 2A and B). We believe that the negative effects of PU139 on the *Smp14* transcriptional activity can be attributed to the inhibition of histone acetyltransferase activities and not to the alteration in coactivator or transcription factor expression levels, transcription factor binding to the DNA or coactivator recruitment. Therefore, we performed qPCR analysis of the SmRXR1 and SmNR1 transcription factors and of the SmGCN5 and SmCBP1 coactivators. As shown in Fig. S2, the PU139 treatment did not affect the mRNA levels of these transcription factor or coactivator targets. From the data presented in Fig. 2, we concluded that the deregulation of chromatin acetylation directly affects Smp14 production.

### The HAT inhibitor PU139 prevents chromatin decondensation at the *Smp14* promoter

**Figure 3:**
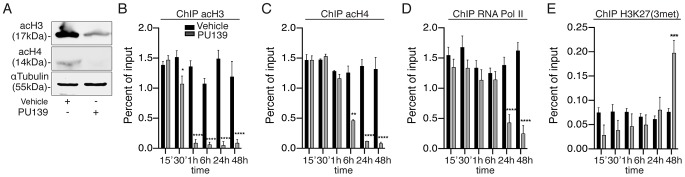
PU139 can reverse histone acetylation, keeping the *Smp14* promoter in a repressed state. Ten adult worm pairs were cultivated with 20 µM PU139 or vehicle for 48 h. (A) Western blot analysis of total worm extract. The levels of *S. mansoni* acetylated histone H3 and H4 are shown. (B–E) ChIP analysis of *S. mansoni* histone modifications at the *Smp14* promoter. Fifty worm pairs were treated with 20 µM PU139 or vehicle at different time points and submitted to chromatin extraction. Chromatin was immunoprecipitated with antibodies directed against acetylated H3 and H4, RNA pol II (markers of transcriptionally active chromatin) and H3K27me3 (a marker of repressed chromatin). ChIP DNA (*Smp14* promoter) was quantified by real-time PCR and normalized as a percentage of input DNA. Results are pooled from four independent experiments. Student's t-test was applied, with *p<0.05, ***p<0.001 and ****p<0.0001.

Based on the data presented above, we analyzed the acetylation status of the chromatin at the *Smp14* promoter site. First, we evaluated the overall acetylation levels of histones H3 and H4, which are substrates of SmGCN5 and SmCBP1 [9], [11], in the worm pairs cultivated for two days with or without the HAT inhibitor PU139. The Western blot analysis demonstrated that histones H3 and H4 were acetylated in the worms treated with the vehicle, and H3 was highly acetylated (Fig. 3A). Importantly, the worms treated with PU139 demonstrated a significant reduction in H3 and H4 acetylation (Fig. 3A). Chromatin loci undergoing transcriptional activation are known to exhibit an enhanced activator-dependent recruitment of the HAT machinery [17], [18]. We therefore performed ChIP assays (Fig. 3B–E) to determine the histone acetylation status at the *Smp14* promoter site. ChIP was performed using the chromatin prepared from the worm pairs cultivated for up to 48 h in the presence of vehicle or PU139. The results (Fig. 3B and C) suggested that schistosome HATs occupied the *Smp14* promoter site and maintained the acetylation of H3 and H4 *in vivo* (black bars). The *Smp14* promoter was co-immunoprecipitated by the anti-RNA polymerase II antibody (Fig. 3D, black bars), indicating that the *Smp*14 gene was transcribed. Most importantly, when we analyzed the immunoprecipitated chromatin from PU139-treated worms, we observed a significant decrease in the levels of acetylated H3 or H4 (Fig. 3B and C, gray bars). These data suggested that the histone hypo-acetylation at the *Smp14* promoter site resulted in an inactive, repressed chromatin state. Indeed, the lack of RNA polymerase II recruitment to the *Smp14* promoter loci (Fig. 3D, gray bars) suggested that the *Smp*14 gene was inactivated. We next confirmed that the *Smp14* promoter assumed a transcriptionally inactive configuration through the immunoprecipitation of the chromatin using the anti-H3K27me3 antibody (Fig. 3E, 48 h), a known biomarker of repressed chromatin. The data (Fig. 3) also suggested that although the PU139 treatment altered the levels of schistosome chromatin acetylation as early as 30 min after the initial exposure for H3 (Fig. 3B) and 6 h for H4 (Fig. 3C), the inactivation of the *Smp14* promoter was achieved after 48 h of histone acetylation inhibition. These data are in agreement with the data shown in Fig. 2.

### HAT inhibition impairs the production of normal *S. mansoni* eggs

**Figure 4:**
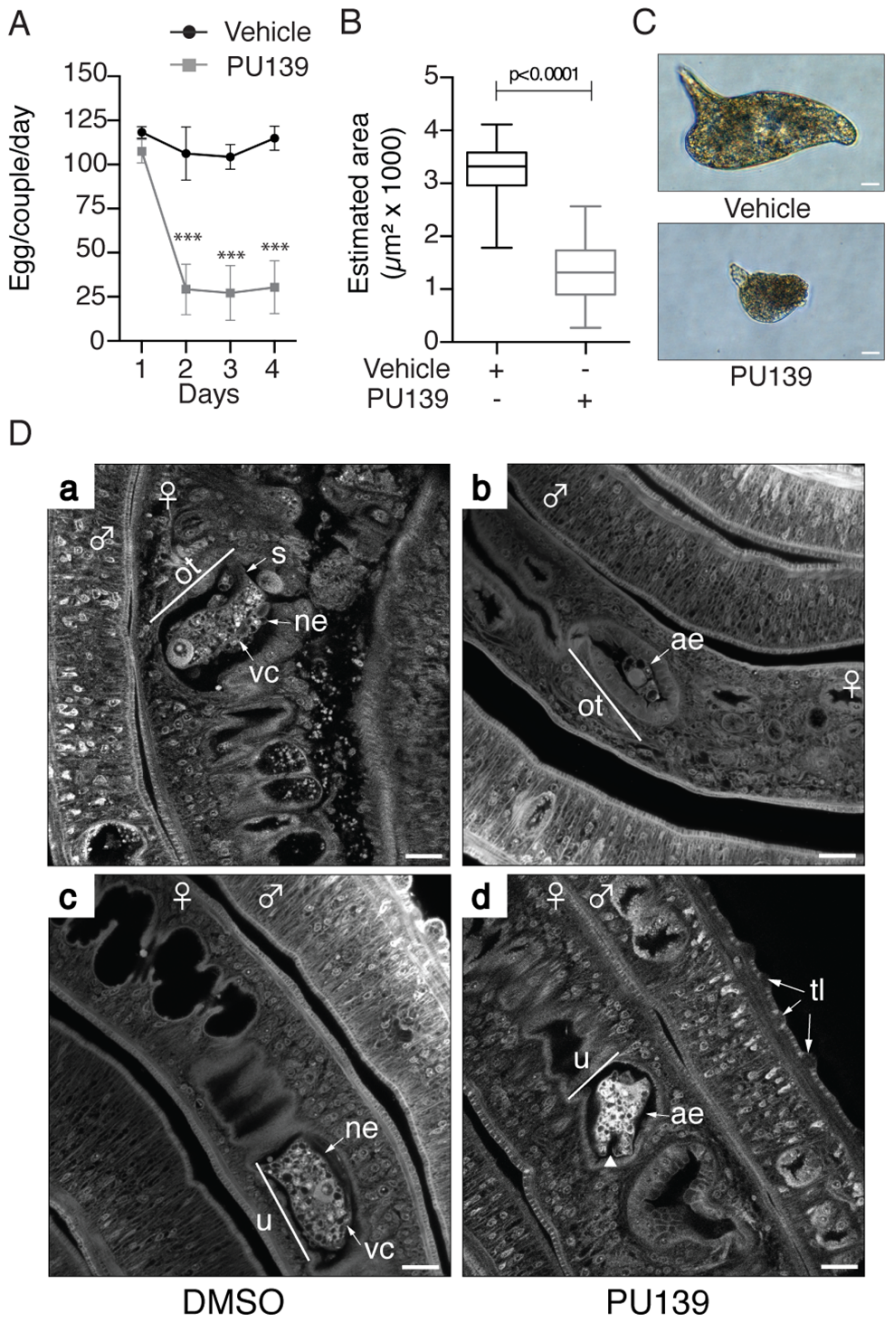
HAT inhibition affects the normal development of *S. mansoni* eggs. Ten adult worm pairs were cultivated with 20 µM PU139 or vehicle up to four days, and the number of eggs was counted on a daily basis (A). The estimated areas (length and width) of these same eggs were measured (B). Student's t-test was applied, with ***p<0.001. The morphology of the eggs was analyzed under optical microscopy. Scale bars: 10 µm (C). Adult worm pairs under the same treatment were fixed and stained with hydrochloric carmine for confocal laser scanning microscopy (CLSM) analysis. ne: normal egg; ae: abnormal egg; ot: ootype; vc: vitelline cells; u: uterus. The arrowhead in “ae” points to a fissure. tt: tegument tubercles. Scale bars: 20 µm (D).

**Figure 5:**
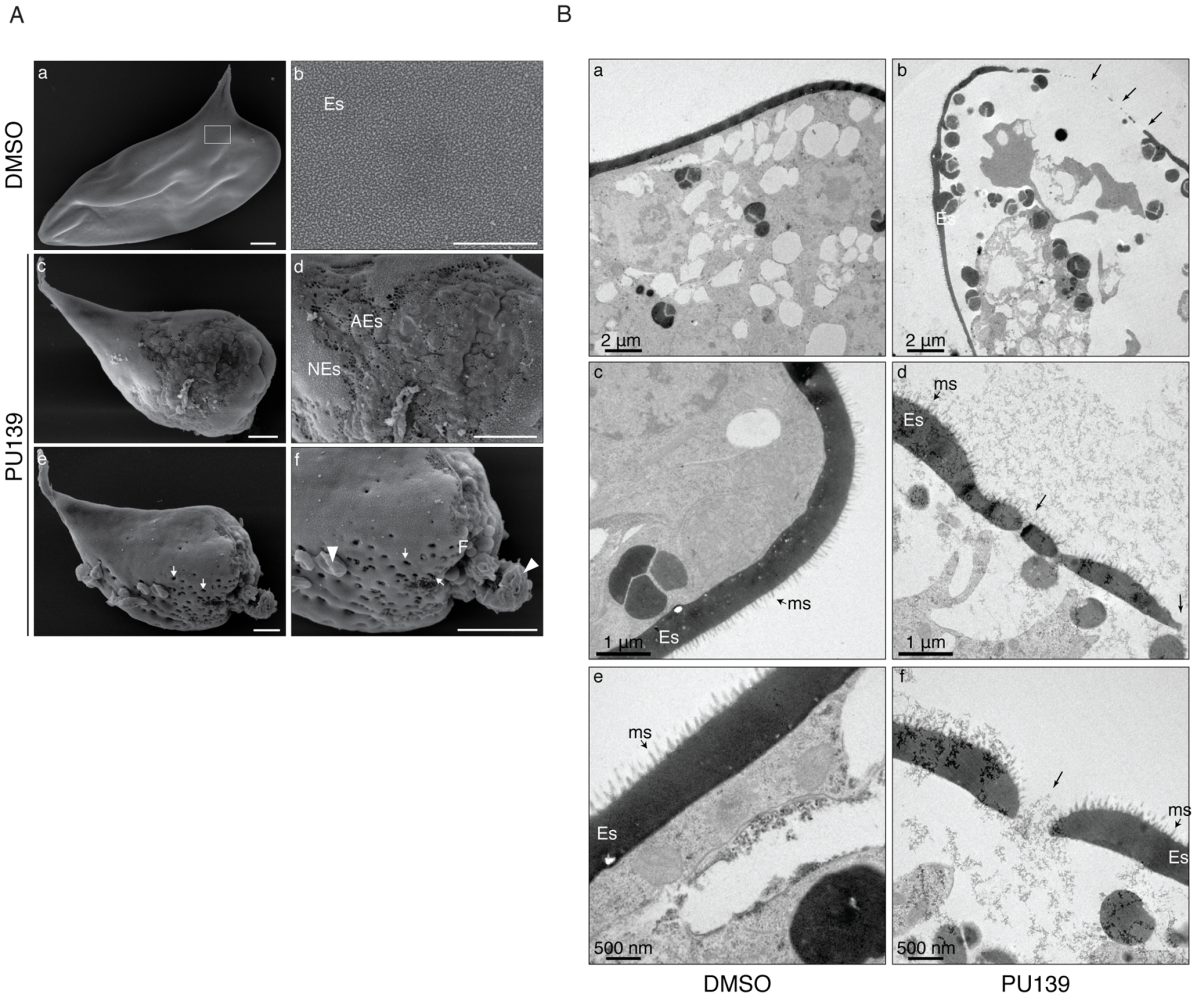
HAT inhibition compromises eggshell integrity. (A) Scanning electron microscopy of eggs laid by worm pairs cultivated for two days with vehicle (panels a and b) or 20 µM PU139 (panels c, d, e and f). A normal *S. mansoni* egg is shown (panel a) with the typical smooth coat of microspines on the eggshell (Es, panel b) seen at higher magnification (boxed area in panel a). PU139 treatment severely affected eggshell formation and integrity (panel c). Gross structural defects on the eggshell can be visualized at higher magnification (panel d; NEs: normal eggshell, AEs: abnormal eggshell). A remarkable phenotypic defect observed in eggs laid by PU139-treated parasites was the presence of holes in the eggshell (arrows in panel e). At a higher magnification, it can be clearly observed that, besides the holes (panel e and f, arrows), a large fissure in the eggshell was formed (panel f, F), leading to leakage of egg contents (panel f, arrowheads). Scale bars 10 µm. (B). Transmission electron microscopy of eggs laid by worm pairs cultivated for two days with vehicle (panels a, c and e) or 20 µM PU139 (panels b, d, and f). Normal eggs (panels a, d and e) show a thick and continuous eggshell (Es). The characteristic microspines (ms) of *S. mansoni* eggs are indicated by the arrows. The eggshells of the eggs laid by PU139-treated females (panels b, d and f) revealed remarkable differences in their structure, when compared with the control eggs, showing much thinner and discontinuous (arrows) eggshells. The holes in the eggshells were confirmed by TEM.

The data presented in Figs. 1, 2 and 3 demonstrated clearly that histone acetylation plays a key role in Smp14 expression. Because Smp14 is a major eggshell component, we reasoned that the inhibition of histone acetylation would affect both egg production and structure. The worm pairs cultivated in the presence of 20 µM PU139 for two days laid significantly fewer eggs compared with the controls (Fig. 4A). A more detailed analysis revealed that the eggs were much smaller (Fig. 4B) and exhibited morphological defects, including invaginations of the egg surface and abnormal lateral spines (Fig. 4C, lower panel). Confocal laser scanning microscopy (Fig. 4D) demonstrated that the eggs inside the ootype (ot) of the paired female worms treated with vehicle were typical, normal eggs (Fig. 4D, panel a, ne) with a fully developed lateral spine (s) and several vitelline cells (vc). Control worms also showed a healthy egg inside the uterus, ready to be laid (Fig. 4D, panel c). In contrast, when the paired females incubated with PU139 were analyzed, we observed an undeveloped egg within the ootype (Fig. 4D, panel b, ae). That the PU139 treatment indeed affected egg development could be confirmed by the observation that the egg inside the uterus is malformed, and with a compromised eggshell (Fig. 4D, panel d, arrowhead). The normal physiology of the paired male worms was evaluated by the integrity of the tegumental tubercles (tt). Notably, the culture conditions used, including the presence of vehicle or PU139, did not change the worm behavior, i.e., they remained active and paired (Fig. 4D), or alter their morphology (data not shown). To confirm that the negative effects on the eggs (Fig. 4) were caused by the disruption of the eggshell integrity, we used scanning electron microscopy to examine the eggshell. As expected, the eggshells from the vehicle treatment group exhibited the typical smooth coat (Fig. 5A, panel a) and surface microspines (Fig. 5A, panel b refers to the boxed area in panel “a” at a higher magnification). Strikingly, the eggs from the PU139-treated parasites were much smaller and exhibited severe eggshell defects (panels c, d, e and f), including holes (panels e and f, arrows) and a large fissure (panel f, line), which ultimately led to the leakage of egg contents (panel f, arrowheads) [19], [20]. To analyze these eggshells in more detail, we made use of transmission electron microscopy (Fig. 5B). Normal eggs (panels a, d and e) show thick and continuous eggshells (Es). The characteristic microspines (ms) of *S. mansoni* eggs are indicated by arrows. When we analyzed the eggs laid by PU139-treated females (Fig. 5B, panels b, d and f), remarkable alterations in their eggshells were observed, which were notably much thinner and discontinuous (arrows). The holes in the eggshells could also be visualized in detail (arrows in panels d and f).

### HAT inhibition affects the development of the female reproductive system

**Figure 6:**
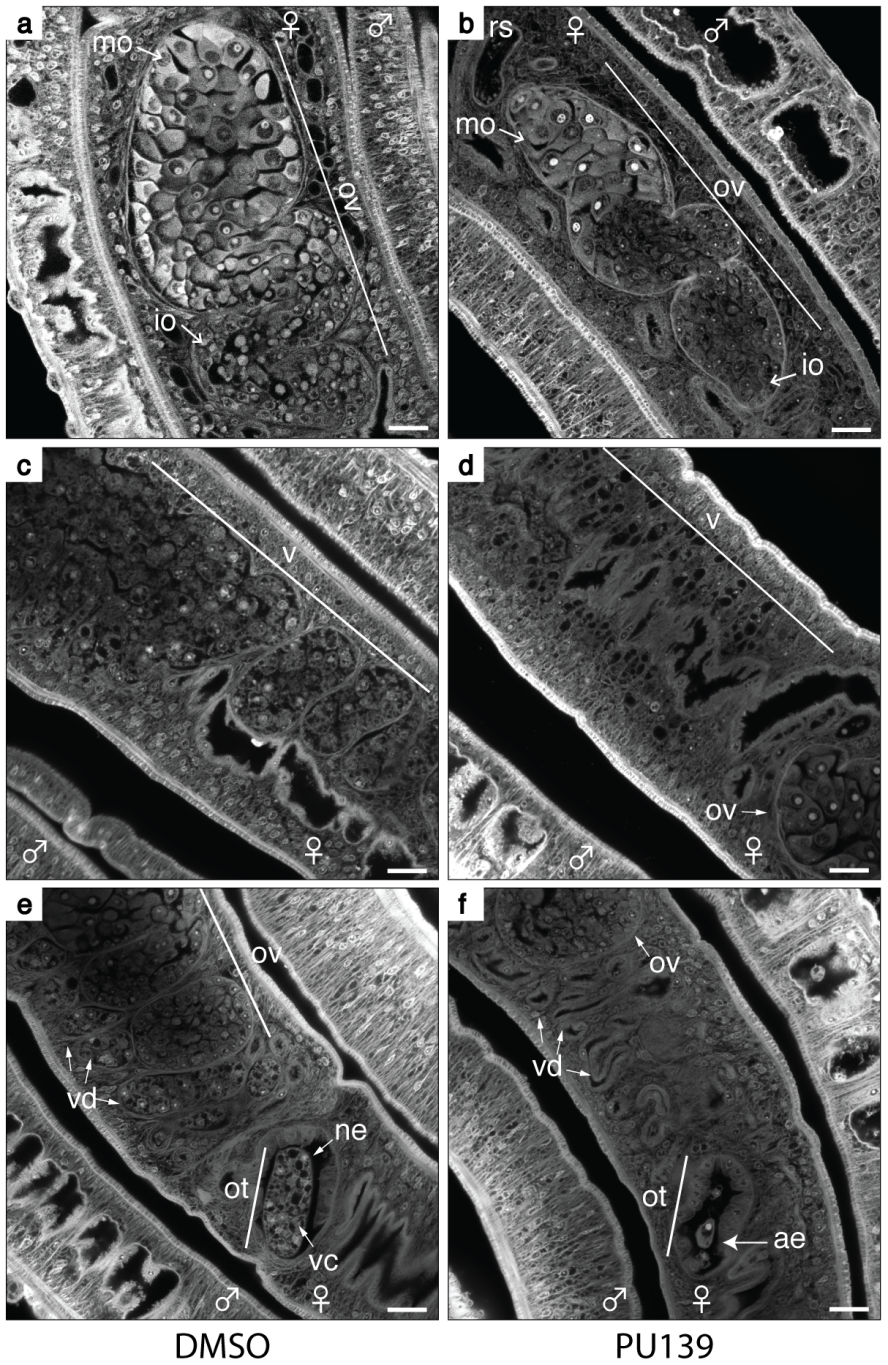
HAT inhibition compromises the reproductive system of female worms. Ten adult worm pairs were cultivated with 20 µM PU139 (panels b, d and f) or vehicle (panels a, c and e) for two days. The worms were fixed and stained with Certistain (Merck) for confocal laser scanning microscopy (CLSM) analysis. ne: normal egg; ae: abnormal egg; ot: ootype; ov: ovary; mo: mature oocytes; io: immature oocytes; vd: vitelline ducts; vc: vitelline cells; rs: receptaculum seminis. The PU139 treatment provoked several negative phenotypes: 1 - the morphology of the ovaries and oocyte maturation (panel b); 2 - the vitellaria size and the number of vitelline cells (panel d); 3 – The vitelline duct and ootype depleted of vitelline cells (panel f). Scale bars: 20 µm.

In the course of our microscopy analysis for the effect of PU139 treatment on egg development, we noticed that the HAT inhibition had a broader negative phenotype on females. When we analysed the ovaries of DMSO-treated females (Fig. 6, panel a), we observed the typical morphology of a healthy ovary, with a large number of immature (io) and mature (mo) oocytes. In contrast, PU139-treated females (Fig. 6, panel b) revealed a smaller ovary, with fewer mature oocytes (compare with panel a).

The importance of the vitellaria in the production of eggshell precursors and nutrients to develop the egg is well known [16]. In the DMSO-treated females, we observed a large and fully developed vitellaria (Fig. 6, panel c), as well as visible vitelline cells within the vitelline ducts (panel e). In contrast, in females treated with PU139, the vitellaria revealed a poriferous structure, with very low numbers of vitelline cells (panel d). In addition, many fewer vitelline cells were observed in the vitelline ducts (panel f).

### SmCBP1 and SmGCN5 are required for *Smp14* expression and egg development

**Figure 7:**
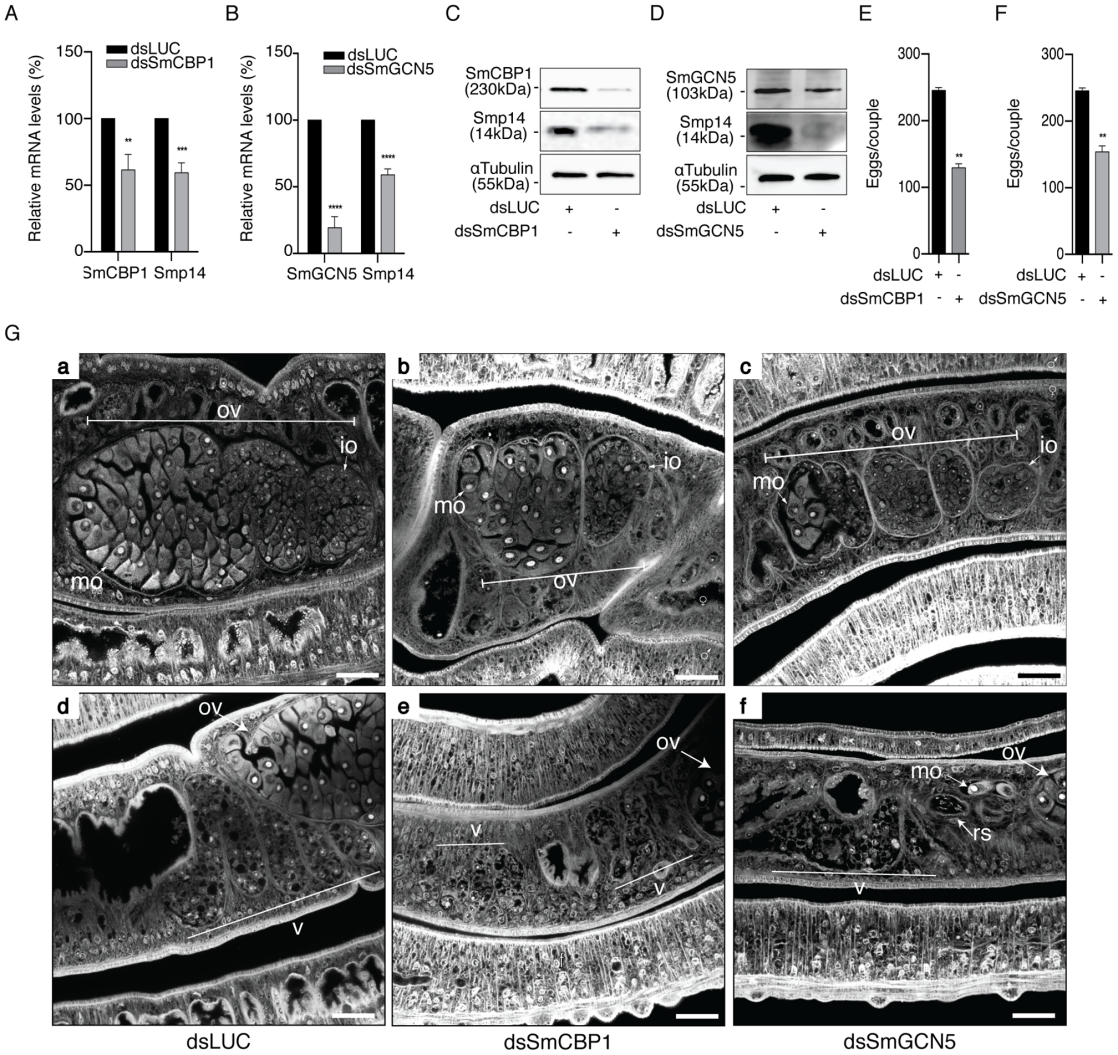
SmCBP1 and SmGCN5 play a key role in Smp14 expression and female sexual reproductive development. A dsRNAi experiment was carried out on sixteen adult worm pairs cultivated for seven days. Worms were electroporated and soaked with dsRNAi from SmCBP1 or SmGCN5 or with the non-specific dsRNAi LUC. On the seventh day of culture, the mRNA levels of SmCBP1, SmGCN5 or *Smp14* (graphs A and B) were determined by qRT-PCR, normalized by the α-tubulin transcript levels. The results are depicted in relation to the non-specific dsRNAi (dsLUC) mRNA levels. The effects of the dsRNAi on targeted-protein synthesis were assessed by Western blot (panels C and D). The total number of eggs laid by the same parasites that received the dsRNAi was counted (graphs E and F). Adult worms from the dsRNAi experiments were analyzed by confocal laser scanning microscopy (G). Worms received either the control dsRNAi LUC (panels a and d) or the dsRNAi for SmCBP1 (panels b and e) or SmGCN5 (panels c and f). Panels a–c, and d–f, reveal the effect of the dsRNAi in the ovary, and vitellaria, respectively. OV: ovary; mo: mature oocytes; io: immature oocytes; v: vitellaria; rs: receptaculum seminis. Results are pooled from three independent experiments. Western blots were repeated three times. The confocal microscopy images are representative of several parasites analyzed. Scale bars 10 µm. Student's t-test was applied, with *p<0.05, **p<0.01, ***p<0.001 and ****p<0.0001.

To directly correlate the HAT activities of SmGCN5 and SmCBP1 with the regulation of *Smp14* expression and the consequent effects on eggshell formation and egg development, we performed functional dsRNAi assays in adult worm pairs. The mRNA levels of SmCBP1 and SmGCN5 were knocked down 38.4% and 80.5%, respectively (Fig. 7A, SmCBP1; 7B, SmGCN5). Next, we demonstrated that the dsRNAi significantly affected the expression of *Smp14*, which is mediated by SmCBP1 and SmGCN5 (Fig. 7C and D, see protein levels; top panels). Most significantly, the knockdown of both of the schistosome HATs led to an approximate 50% reduction in the transcription of *Smp14* (Fig. 7A and B, *Smp14*) and an effective decrease in the Smp14 protein level (Fig. 7C and D, middle panels). The cultivated worms that received the SmCBP1 or SmGCN5 dsRNAi demonstrated a significant reduction in the number of eggs (Fig. 7E and F) after the first four days in culture. In addition, these eggs exhibited an abnormal morphology, similar to that observed following the PU139 treatment (Fig. 4A). The dsRNAi experiments provided additional confirmation that both SmCBP1 and SmGCN5 play major roles in egg development.

Not surprisingly, the knockdown of SmCBP1 and SmGCN5 also affected the reproductive system of the female worms (Fig. 7G), illustrated by the decrease in ovary size and the number and developmental stage of the oocytes produced (panel b for SmCBP1, and panel c, for SmGCN5). As expected, partial depletion of SmCBP1 (panel e) or SmGCN5 (panel f), also affected the vitellaria of females, visible by their reduced size, with fewer vitelline cells, when compared to the control RNAi (panel d). However, upon inspection of the male worm reproductive system, no apparent negative phenotype was observed; the male worms had fully developed testicular lobes containing plenty of germinal cells (Fig. S3B). Nevertheless, it is important to point out that we do not exclude the possibility of a negative effect of HAT inhibitors in males. We believe that mature females are readily affected due to their high metabolic rate required for sexual maturation and egg production. Thus, it is likely that males that were exposed to longer incubation periods in the presence of PU139 would also be affected. It is expected then, that female and male worms would undoubtedly experience a general transcriptional deregulation upon inhibition of their two main HAT enzymes, which would result in various physiological disorders.

## Discussion

Schistosomiasis, which is caused by the blood fluke *S. mansoni*, has afflicted humans for thousands of years, and represents a major and still largely unacknowledged burden [1], particularly in sub-Saharan Africa where about 90% of infected individuals live [21]. In the absence of an effective prophylactic vaccine, a viable approach to controlling the disease has been the preventative mass-treatment of human populations in endemic areas using Praziquantel, the only effective drug available [2]. However, Praziquantel, although safe, effective and cheap is far from a perfect drug. First, it is not active against the juvenile parasites; second, the mechanism of action is not known, which limits the development of improved derivatives; and third, most concerning, Praziquantel-resistant parasites may eventually prevent the continued use of the drug [2] particularly in view of its massive use in mass chemotherapy campaigns [22]. Therefore, there is an urgent need to identify new parasite targets for the development of novel therapeutics.

Epigenetic targets, including enzymes that introduce histone modifications affecting gene transcription, are particularly attractive because these enzymes are already targeted in a variety of pathologies, particularly cancer [23] and a large number of drug candidates have been developed that can serve as the basis for developing selective inhibitors of schistosome enzymes.

Sexually mature female schistosomes produce 200–300 eggs daily, and the eggs constitute key components of the transmission and immunopathology of schistosomiasis. We have long been interested in understanding the molecular mechanisms involved in egg development, with the objective of finding the means to block egg production. Therefore, we have focused our studies on the mechanisms that regulate the expression of the major *S. mansoni* eggshell gene, *Smp14*. Our previous research suggested that *Smp14* transcription is regulated by nuclear hormone receptors and coactivators that contain histone-modifying activities [9]–[14], such as acetylation. The two main *S. mansoni* HATs, SmCBP1 and SmGCN5, have been suggested to act as transcription activators [11]–[13]. Importantly, SmGCN5 has been directly linked to *Smp14* activation *in vivo* via the localization in the nuclei of mature vitelline cells [12] where the Smp14 protein is produced [24]. SmGCN5 contains two unique sequences immediately flanking its catalytic domain [9] that are not found in any other GCN5 orthologues. This distinction might be targeted in novel therapeutics directed against this parasite.

Cellular homeostasis in eukaryotes is regulated by a fine balance between histone acetylation and deacetylation. Any loss of HAT and HDAC function via mutation or inhibition generally results in a disease state. As an example, the imbalance in histone acetylation activity mediated by HAT inhibitors compromises the development of protozoan parasites [25], [26]. In the specific case of *S. mansoni*, recent studies [27]–[29] demonstrated the feasibility of targeting histone deacetylation activity as a promising intervention strategy.

To demonstrate the relationship between histone acetylation and egg development, we investigated the effect of inhibitors of human HATs (Table S1) on the activities of SmCBP1 and SmGCN5. The inhibition of the acetyltransferase activities of SmCBP1 and SmGCN5, along with the concomitant inactivation of *Smp14* expression, would be proof of concept for the inhibition of histone acetylation in female schistosomes as a feasible strategy for controlling eggshell formation.

**Figure 8:**
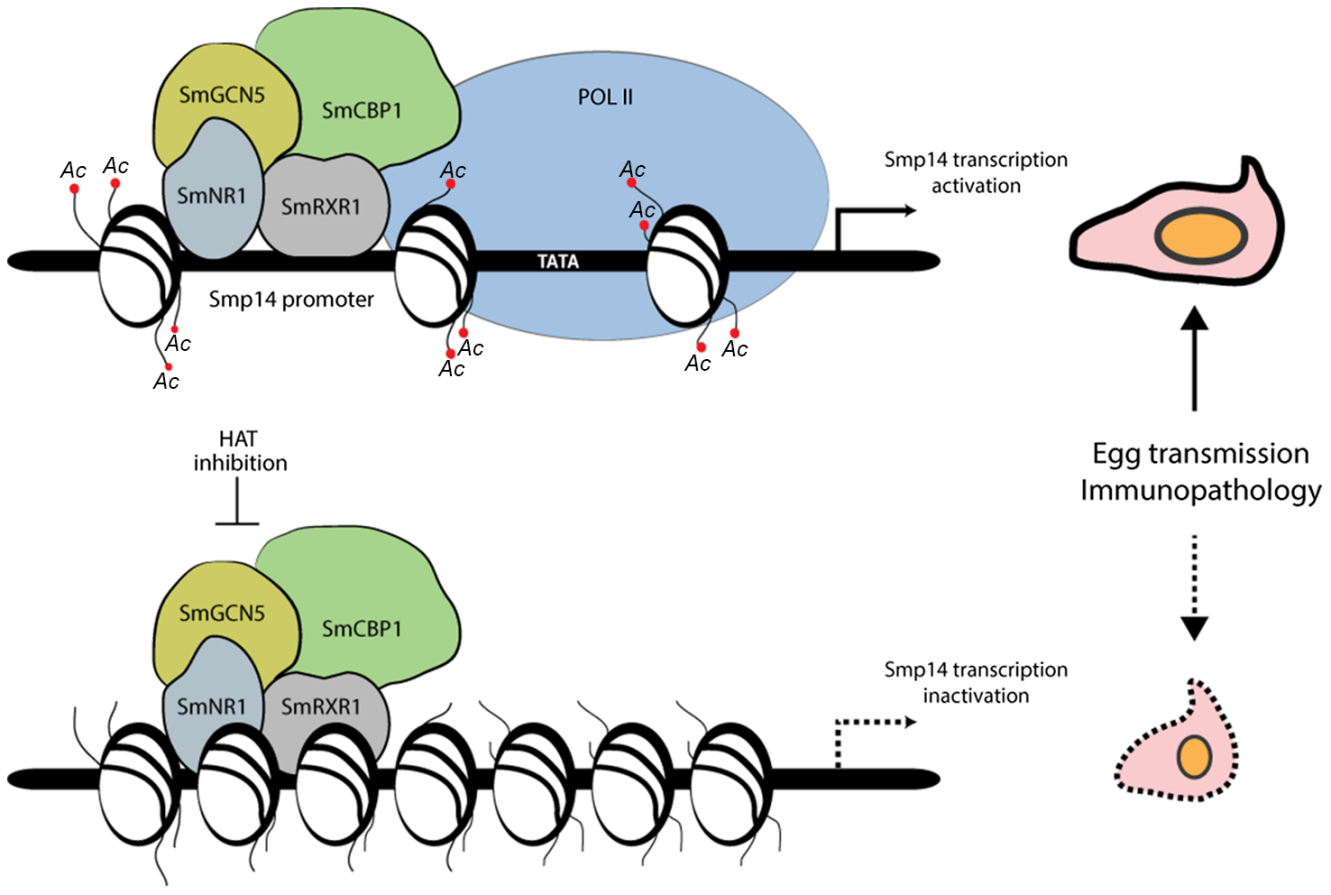
Proposed model of the acetylation-dependent activation of *Smp14* transcription and eggshell formation. The nuclear receptor heterodimer SmRXR1/SmNR1 binds to a specific DNA response element present in the *Smp14* promoter [14] and recruits the two histone acetyltransferases SmGCN5 and SmCBP1. Chromatin is then remodeled, RNA Pol II is attracted to the promoter and transcription occurs. Synthesis of Smp14 proteins will lead to eggshell formation, which is a prerequisite for egg dissemination and granuloma formation. Interfering with the histone acetyltransferase activities of either SmCBP1 or SmGCN5 reverses chromatin decondensation, with no access for RNA Poll II. Transcription of the *Smp14* gene is halted, compromising eggshell integrity, which can ultimately lead to a reduction in the transmission and immunopathology of schistosomiasis.

The data presented in this study clearly demonstrate that the *in vivo* transcription of *Smp14* requires nuclear receptor signaling, including the recruitment of coactivators with HAT activities (Fig. 8). Despite the fact that *S. mansoni* expresses six families of proteins with HAT activities [30], we hypothesized that SmCBP1 and SmGCN5 are the most important for *Smp14* transcriptional activation. HAT1B and Tip60, members of the HAT family in *S. mansoni*, did not interact with SmRXR1 or SmNR1 *in vitro*, although both exhibited HAT activities (data not shown). Surprisingly, the *S. mansoni* genome does not present genes encoding members of the p160 HAT coactivators [31]. In other species, the p160 HAT coactivators are the first to recognize the nuclear receptors, followed by the recruitment of other HATs and the RNA Pol II complex [31]. Because of this distinction, we believe that SmGCN5 and/or SmCBP1 act as the main platform for the assembly of the transcriptional initiation complex on the *Smp14* promoter.

If SmCBP1 and SmGCN5 are the key coactivators of *Smp14* expression, then the inhibition of these enzymes should have a direct effect on egg development *in vivo*. Indeed, this study demonstrated that the targeted inhibition of the catalytic domains of the schistosome HATs by PU139 treatment or the disruption of HAT transcription/translation by dsRNAi significantly decreased the Smp14 mRNA and protein levels, culminating in gross morphological eggshell defects and hindering egg development (Fig. 8). Interestingly, a similar phenotype in the *S. mansoni* eggshell was observed previously when two tyrosinases were inhibited by kojic acid [19]. These schistosome tyrosinases catalyze the cross-linking of tyrosine-rich Smp14 proteins, which is the final step in eggshell hardening [32]. Notably, other studies have contributed to the current understanding of *S. mansoni* egg development and have suggested potential targets for schistosome management [16], [33]–[35]. For example, Herbimycin A, an inhibitor of protein tyrosine kinases, blocked mitotic activity and egg production in paired females [33]. Recently, the same authors suggested that mitosis and eggshell formation were controlled via the cooperation of the Src-kinase and TGFβ receptor pathways [35]. Previous studies demonstrated that Herbimycin A decreased the mRNA levels of a variety of different genes, including genes that encode eggshell proteins. Although the molecular mechanism(s) involved in the regulation of eggshell genes by Src-kinase and the TGFβ receptor has not been elucidated, it is possible that tyrosine phosphorylation plays a role in the activation of the nuclear receptor heterodimer SmRXR1/SmNR1. SmRXR1/SmNR1 ligands have not been identified. However, the ligand-independent activation of nuclear receptors has been attributed to the phosphorylation of the AF-1 activation domain [36], [37]. Moreover, it is known that neither SmRXR1 nor SmNR1 binds retinoic acid or 9-*cis*-retinoic acid *in vitro* [13].

Modulators of enzyme activity are attractive agents for deciphering enzyme function [22]. Although silencing studies are used extensively to understand protein function, the lack of a particular protein in a specific cell type might lead to aberrant signaling and spurious conclusions. The modulator-based approach affects only the functional activity, enabling more precise conclusions regarding protein function, distinct from protein expression. In this study, we applied both approaches and concluded that histone acetylation by SmCBP1 and SmGCN5 creates the required epigenetic state for *Smp14* transcriptional activation and eggshell formation. Our findings will contribute not only to a better understanding of sex- and tissue-specific gene regulation in *S. mansoni* but also provide an alternative strategy for interfering with egg production.

## Materials and Methods

### Ethics statement

The experiments involving hamsters as the final hosts to maintain the schistosome life cycle were performed in accordance with the European Convention for the Protection of Vertebrate Animals used for Experimental and other Scientific Purposes (ETS No. 123; revised Appendix A) and were approved by the committee for ethics in animal experimentation of the Nord-Pas de Calais region (Authorization No. AF/2009) and the Pasteur Institute of Lille (Agreement No. A59-35009).

### Parasite stock

A Puerto-Rican isolate of *S. mansoni* was maintained in *Biomphalaria glabrata* as the intermediate host and in Syrian hamsters (*Mesocricetus auratus*) as the definitive host [38] The adult worms were obtained by hepatoportal perfusion at 42–49 days post-infection.

### Plasmids and gene reporter assay

The full-length cDNA of SmGCN5 [9] was cloned into pcDNA 3.1 A(+) (Invitrogen). The target sequence of the Smp14 promoter [5] (position −400 to −369 bp from the transcription start site, repeated three times) was cloned into pGL4.23 (Promega) upstream of the luciferase reporter gene, and the construct was named Smp14/3X-pGL4.23. The full-length SmCBP1-pcDNA 3.1 A(+) has been described previously [11]. The SmNR1-pcDNA 3.1 A(+) and SmRXR1-pcDNA 3.1 A(+) were a kind gift from Pr. Philip T. LoVerde (University of Texas, San Antonio). The oligonucleotides used in this study are described in Table S2. The HEK293 cells were maintained in DMEM supplemented with 10% fetal bovine serum at 37°C with 5% CO_2_. The cells were plated one day before transfection in 24-well plates at a density of 80,000 cells per well. The transfections were performed using 150 ng of each plasmid (Smp14/3X-pGL4.23, pGL4.74, SmCBP1-pcDNA, SmGCN5-pcDNA, SmNR1-pcDNA, SmRXR1-pcDNA and/or empty pcDNA3.1A). The cells were lysed 24 h after transfection, and the analysis of luminescence as a measure of luciferase activity was performed (dual-luciferase reporter assay system, Promega). The sodium butyrate (NaB), trichostatin A (TSA) or HAT inhibitors were added to the plated cells at 6 hours after transfection. MTT and LDH assays were used to measure the cell viability (data not shown). Statistical significance was determined using Student's t-test.

### Schistosome *in vit*ro culture, inhibitor treatment and microscopy analysis

The adult worms were obtained by whole-body perfusion of hamsters following a 6-week infection, and the worms were cultivated as previously reported [38]. For each treatment condition, 10 worm pairs were maintained in 60-mm diameter culture dishes in 2 mL of culture medium, supplemented with 10 nM to 50 µM PU139. The medium and the HAT inhibitor PU139 [15] were refreshed every 24 h during the treatment period (1–4 days). After treatment, the total RNA was extracted, and qRT-PCR was performed for the target genes (the oligonucleotides are listed in Table S2). The Western blots were performed using antibodies directed against acetylated histones H3 and H4 (Upstate), Smp14 (polyclonal serum, a kind gift from Pr. Philip T. LoVerde) and human α-tubulin (Abcam). For the microscopic analysis, the adult worms were fixed and stained as previously described [39]. Confocal scanning laser microscopy was performed on a Zeiss LSM 710 microscope equipped with a 488-nm HE/Ne laser and a 470-nm long-pass filter but without the reflection mode.

The eggs were counted and photographed every 24 h, and the worm motility and morphology were monitored daily. Approximately 300 eggs from three independent experiments were measured (length and width) as previously reported [20]. Approximately 200 eggs pooled from three independent experiments were fixed in 2.5% glutaraldehyde; 4% freshly prepared formaldehyde in 0.1 M cacodylate buffer, pH 7.2, for 1 h at room temperature and maintained at 4°C. The samples were then subsequently washed in 0.1 M cacodylate buffer, pH 7.2 and only the eggs were adhered to the coverslips coated with 1% gelatin. Samples were post-fixed in 1% OsO_4_ and 0.8% K_3_Fe (CN)_6_; washed in 0.1 M cacodylate buffer, pH 7.2; dehydrated in a graded ethanol series (20°–100° GL) for one hour each step; critical-point dried in CO_2_; mounted on metallic stubs and coated with gold (20–25 nm deposited). The samples were examined under FEI Quanta 250 scanning electron microscope, operating at 15–20 kV and a pressure of 2–8×10^−4^ Pa. Transmission electron microscopy was performed on a Tecnai G2 microscope (FEI Company, Eindhoven). Eggs (chemical fixed) were washed in 0.1 M cacodylate buffer, pH 7.2; post-fixed in 1% OsO_4_ and 0.8% K_3_Fe (CN)_6_; washed in 0.1 M cacodylate buffer, pH 7.2; dehydrated in a graded acetone series (20°–100° GL) for one hour each step and embedded in Polybed 812 epoxide resin. Thin-sections (60 nm) were collected on copper grids, stained for 30 minutes in 5% aqueous uranyl acetate, for 5 minutes in lead citrate.

### Chromatin immunoprecipitation (ChIP)

ChIP analysis was performed following an adapted protocol for schistosomes, as previously reported [40]. Briefly, control and PU139-treated parasites were fixed in 1% formaldehyde and sonicated on a Bioruptor UCD-300 (Diagenode) to fragment the chromatin. The immunoprecipitation was performed using rabbit antibodies raised against acetylated histones H3 or H4 (ChIP grade, Upstate), RNA Pol II (ChIP grade, Abcam) or tri-methylated lysine 27 of histone H3 (H3K27-3met, ChIP grade, Abcam), SmNR1, SmRXR1, SmCBP1 and SmGCN5 (Rhea Biotechnology). Non-immunized rabbit serum (Sigma) was used as the control. The precipitated DNA was purified and quantified by qPCR or conventional PCR, using the oligonucleotides described in Table S2. The data were expressed as the percentage of the input DNA not subjected to immunoprecipitation.

### Double-stranded RNAi

In preparation for the double-stranded RNA (dsRNA) synthesis, 500-bp fragments of the firefly luciferase (dsLUC, negative control), SmCBP1 or SmGCN5 cDNAs were amplified by PCR using the gene-specific primers listed in Table S2. The dsRNAs were synthesized in accordance with the manufacturer's instructions (MEGAscript RNA transcription kit, Ambion). Annealing and the integrity of the dsRNAs were confirmed using agarose gel electrophoresis. After electroporation, as previously reported [39], the worms were cultivated for seven days, and the first medium change was performed after the second day. Thereafter, the medium was refreshed every 24 h. The eggs were counted and the worm motility and morphology were monitored daily. For the Western blot analysis, an additional protein extraction step was performed using the RiboPure kit (Ambion) protocol. The SmCBP1 and SmGCN5 polyclonal antibodies were obtained from Rhea Biotech (Brazil). Both the control worms and worms that received dsRNAi were analyzed by confocal scanning laser microscopy.

## Supporting Information

Figure S1 ***Smp14*** **promoter occupancy by schistosome nuclear receptors and HAT coactivators.** ChIP analysis of fifty adult worms freshly perfused. Chromatin was extracted and immunoprecipitated with antibodies directed against acetylated H3, SmRXR1, SmNR1, SmGCN5 and SmCBP1. ChIP DNA (*Smp14* promoter) was analysed by agarose gel and stained with EtBr.
(TIF)

Figure S2 **PU139 treatment does not interfere with the mRNA levels of the target genes.** The same parasites that were cultivated in the presence of PU139 or vehicle (DMSO) and evaluated for Smp14 expression (Fig. 1), egg number and morphology (Fig. 3) were also tested for the mRNA expression of SmRXR1 (A), SmNR1 (B), SmCBP1 (C) and SmGCN5 (D) using qRT-PCR. The data shown represent three independent experiments. Student's t-test was applied, and no statistical significance was observed. The PU139 treatment groups are indicated with black bars, and the DMSO treatment groups are indicated by gray bars.
(TIF)

Figure S3 **Inhibition or partial depletion of histone acetyltransferases do not compromise the** ***S. mansoni*** **male reproductive system.** (A) Adult worm pairs were cultivated for two days in the presence of PU139 or vehicle, fixed and analyzed by confocal laser scanning microscopy. Note that even after treatment, the male and female worms remained paired (A, both panels). Scale bars: 10 µm. (B) Adult worm pairs that received dsRNAs for LUC, SmCBP1 or SmGCN5 were cultivated for seven days and analyzed by confocal laser scanning microscopy. Details of the testicular lobes (tl) are shown in all panels. Scale bars: 20 µm.
(TIF)

Table S1 **Five small synthetic pyridoisothiazolones were selected to test against members of the histone acetyltransferase (HAT) family (PCAF, GCN5, CBP and p300) using histone H3 (amino acid residues 1–21) as a substrate.** The data represent the IC_50_ value [µM] ± standard error. * Numbers in parentheses refer to compound numbering in the reference that describes the structure and synthesis.
(DOCX)

Table S2 **List of oligonucleotides used in the study.** For the ChIP analysis, three regions of the Smp14 promoter were amplified using the referenced primers. For ChIP, the *Smp*14 gene ATP region comprised the sequence from −28 to the ATG initiation codon (+70); the *Smp*14 gene DR17 region comprised the sequence from −480 to −398 and contained a hormone response element; and *Smp*14 gene DR5 region comprised the sequence from −210 to −113 and contained a second hormone response element. For the RNAi experiments, the oligonucleotides contained the T7 promoter sequence (underlined). The bolded text indicates the restriction enzyme sites in the SmGCN5 primers.
(DOCX)
