# Supplementary figures and images for: Epigenetic Changes Modulate Schistosome Egg Formation and Are a Novel Target for Reducing Transmission of Schistosomiasis
Source: PLoS Pathog. 2014 May 8;10(5):e1004116. doi: 10.1371/journal.ppat.1004116 (PMC4014452; doi:10.1371/journal.ppat.1004116)

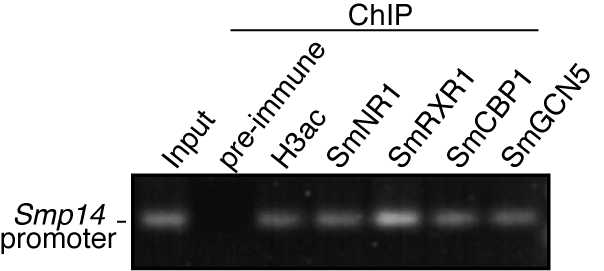

Supplement: Figure S1 — Smp14 promoter occupancy by schistosome nuclear receptors and HAT coactivators. ChIP analysis of fifty adult worms freshly perfused. Chromatin was extracted and immunoprecipitated with antibodies directed against acetylated H3, SmRXR1, SmNR1, SmGCN5 and SmCBP1. ChIP DNA (Smp14 promoter) was analysed by agarose gel and stained with EtBr. (TIF) [file ppat.1004116.s001.tif]

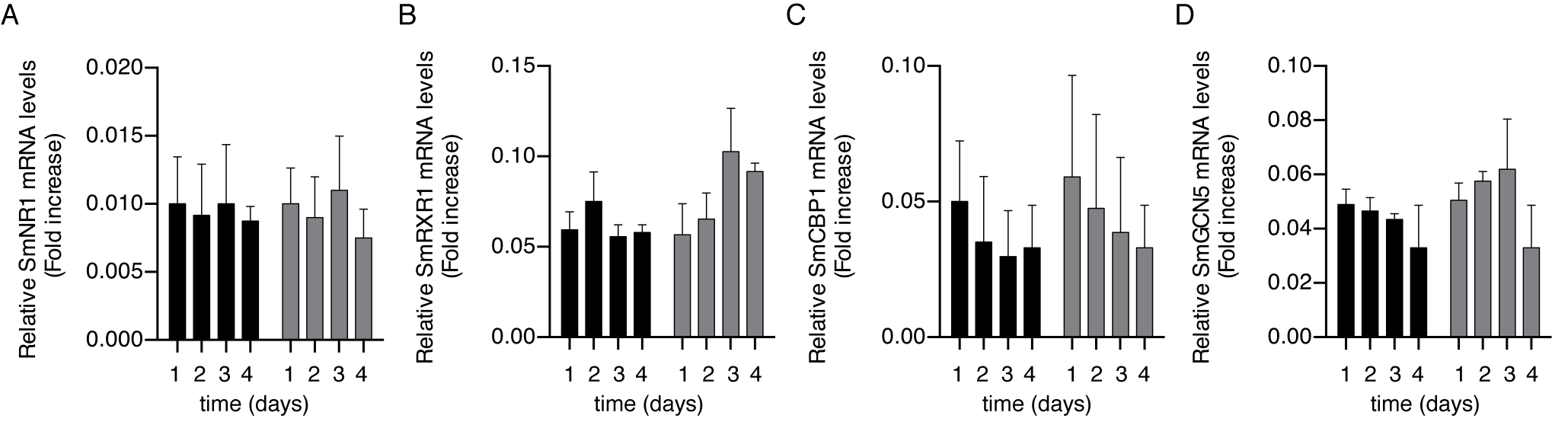

Supplement: Figure S2 — PU139 treatment does not interfere with the mRNA levels of the target genes. The same parasites that were cultivated in the presence of PU139 or vehicle (DMSO) and evaluated for Smp14 expression (Fig. 1), egg number and morphology (Fig. 3) were also tested for the mRNA expression of SmRXR1 (A), SmNR1 (B), SmCBP1 (C) and SmGCN5 (D) using qRT-PCR. The data shown represent three independent experiments. Student's t-test was applied, and no statistical significance was observed. The PU139 treatment groups are indicated with black bars, and the DMSO treatment groups are indicated by gray bars. (TIF) [file ppat.1004116.s002.tif]

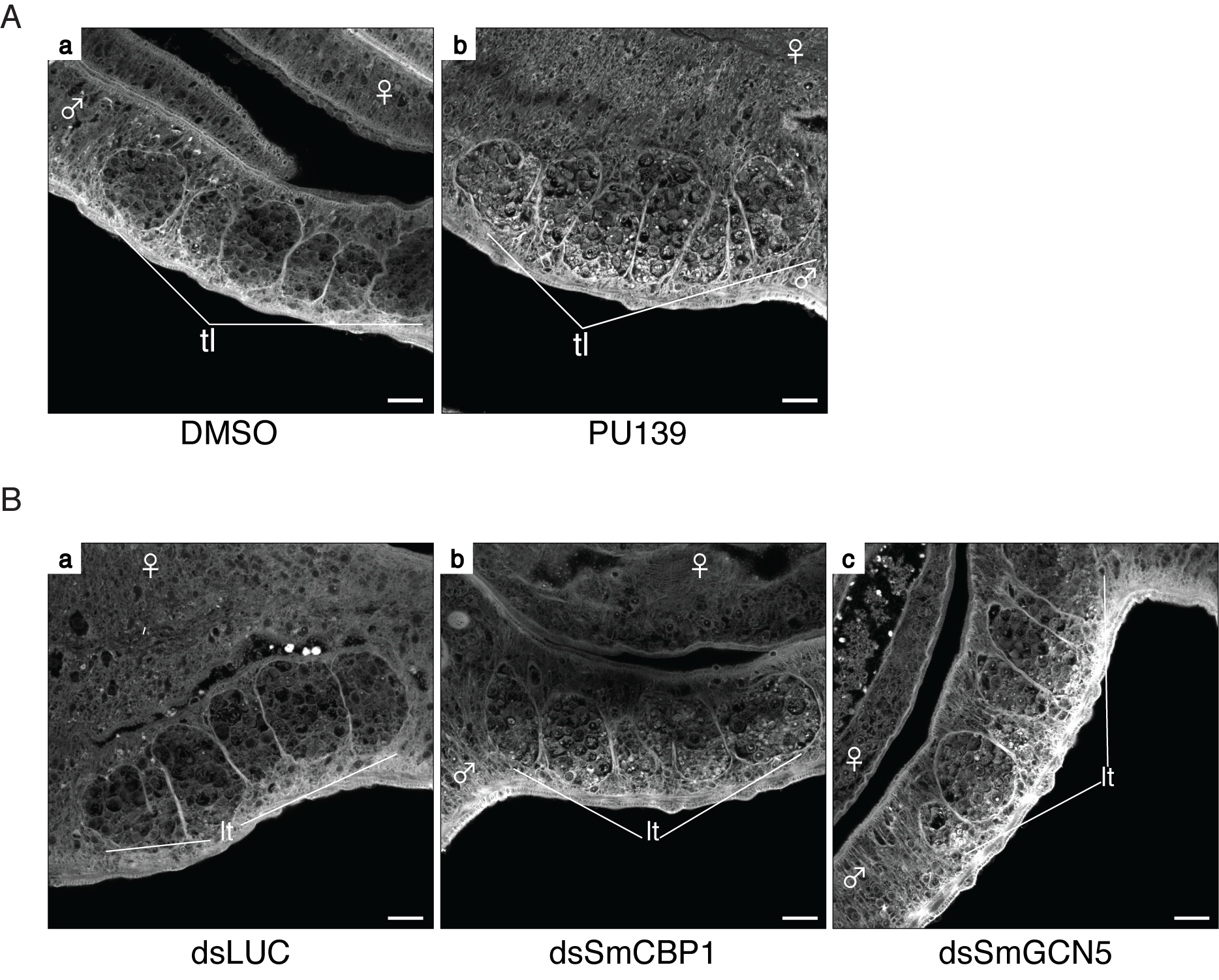

Supplement: Figure S3 — Inhibition or partial depletion of histone acetyltransferases do not compromise the S. mansoni male reproductive system. (A) Adult worm pairs were cultivated for two days in the presence of PU139 or vehicle, fixed and analyzed by confocal laser scanning microscopy. Note that even after treatment, the male and female worms remained paired (A, both panels). Scale bars: 10 µm. (B) Adult worm pairs that received dsRNAs for LUC, SmCBP1 or SmGCN5 were cultivated for seven days and analyzed by confocal laser scanning microscopy. Details of the testicular lobes (tl) are shown in all panels. Scale bars: 20 µm. (TIF) [file ppat.1004116.s003.tif]
