# Supplementary material for: Epigenetic Changes Modulate Schistosome Egg Formation and Are a Novel Target for Reducing Transmission of Schistosomiasis
Source: PLoS Pathog. 2014 May 8;10(5):e1004116. doi: 10.1371/journal.ppat.1004116 (PMC4014452; doi:10.1371/journal.ppat.1004116)
Supplement: Table S1 — Five small synthetic pyridoisothiazolones were selected to test against members of the histone acetyltransferase (HAT) family (PCAF, GCN5, CBP and p300) using histone H3 (amino acid residues 1–21) as a substrate. The data represent the IC50 value [µM] ± standard error. * Numbers in parentheses refer to compound numbering in the reference that describes the structure and synthesis. (DOCX) [file ppat.1004116.s004.docx]

**Pyridoisothiazolone HAT-Inhibitors**

| **Name*** | **CxHx…** | **MW** | **PCAF** *(KAT2B)*  E3_Ch:0509 | **Gcn5** *(KAT2A)*  BPS_100329 | **CBP** *(KAT3A)*  Biomol_T6533 | **p300** *(KAT3B)*  BPS_121001 |
| --- | --- | --- | --- | --- | --- | --- |
| PU139  (4d) | C_12_H_7_FN_2_OS | 246.26 | 9.74 ± 0.24 µM | 8.39 ± 0.22 µM | 2.49 ± 0.09 µM | 5.35 ± 0.36 µM |
| PU141  (8h) | C_14_H_9_F_3_N_2_OS | 310.30 | 130.0 ± 8.49 µM | 87.36 ± 7.93 µM | 2.85 ± 0.20 µM | 5.92 ± 0.17 µM |
| SF7  (4b) | C_12_H_7_ClN_2_OS | 262.72 | 3.53 ± 0.07 µM | 15.47 ± 0.84 µM | 5.28 ± 0.23 µM | 5.71 ± 0.24 µM |
| SF18  (8b) | C_13_H_9_ClN_2_OS | 276.75 | 17.45 ± 1.27 µM | 89.91 ± 2.66 µM | 1.95 ± 0.35 µM | 5.74 ± 0.33 µM |
| SF19  (8c) | C_13_H_8_Cl_2_N_2_OS | 311.19 | 6.94 ± 0.57 µM | 23.70 ± 1.05 µM | 1.27 ± 0.09 µM | 5.32 ± 1.07 µM |
